# Le syndrome de Vogt-Koyanagi Harada dans sa forme purement oculaire: à propos d'un cas

**DOI:** 10.11604/pamj.2014.19.30.5188

**Published:** 2014-09-12

**Authors:** Shamil Louaya, Youssef Bennouk, Mohamed Kriet, Abdelbarre Oubaaz

**Affiliations:** 1 Centre Médico-Chirurgical Agadir, Agadir, Maroc; 2 Hôpital Militaire Avicenne Marrakech, Marrakech, Maroc; 3 Hôpital Militaire d'Instruction Mohamed V Rabat, Rabat, Maroc

**Keywords:** Vogt Koyanagi Harada, décollements séreux rétiniens, pin-points, uvéite, Vogt Koyanagi Harada, serous retinal detachment, pin-points, uveitis

## Abstract

Le syndrome de Vogt-Koyanagi-Harada (VKH) est une affection systémique rare, sévère mettant en jeu le pronostic visuel malgré les traitements agressifs, caractérisée par l'association de plusieurs symptômes: oculaires, méningés, auditifs et cutanés secondaire à une auto-immunisation où le mélanocyte serait la cellule cible. Nous rapportons un nouveau cas rare où l'atteinte oculaire est isolée. La sévérité de l'uvéite réside dans la fréquence des récidives, dans la cortico-résistance et dans la forte incidence des complications qui conditionnent le pronostic et expliquent les acuités visuelles finales médiocres. La prise en charge doit être précoce afin d'essayer d'améliorer le pronostic visuel.

## Introduction

Le syndrome de Vogt-Koyanagi-Harada (VKH) est une affection multi-systémique rare, caractérisée par l'association de plusieurs symptômes: oculaires, méningés, auditifs et cutanés. Ce syndrome revêt trois formes cliniques selon la FIW en 2001: la forme complète, incomplète et possible; cette dernière est exclusivement oculaire tel est notre cas.

## Patient et observation

**Figure 1:**
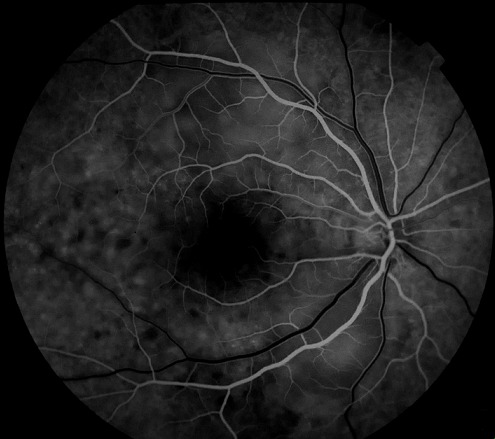
Temps précoces montrant un retard de remplissage choroïdien avec de multiples points hyperfluorescents en tête d’épingle (pin-points)

**Figure 2:**
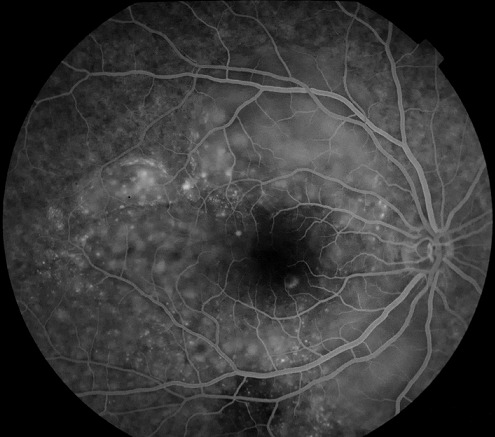
Temps tardifs montrant la confluence des pin-points avec de multiples décollements séreux rétiniens exsudatifs

Nous rapportons Le cas d'un homme de 25 ans, sans antécédents pathologiques ou traumatiques particuliers, présentant une baisse d'acuité visuelle brutale et bilatérale chiffrée à 2 /10° et 1/10° sans autre signe associé avec la présence à l'examen du fond d’œil, des deux yeux, de multiples décollements séreux rétiniens (DSR) maculaires bulleux étendus et à l'angiographie à la fluorescéine, au temps précoces (Figure 1) l'apparition de multiples points hyperfluorescents en tête d’épingle au niveau de l’épithélium pigmentaire; ces points (pin point) augmentent en taille progressivement au cours de la séquence agiographique associés à un aspect hétérogène de la fluorescence choroïdienne qui témoigne d'un trouble de perfusion choroïdienne et au temps tardifs (Figure 2) la confluence de ces pin-points avec accumulation du colorant dans l'espace sous-rétinien délimitant ainsi les multiples décollements séreux rétiniens exsudatifs.

Le bilan biologique, infectieux et immunologique effectué s'est retourné normal. Le diagnostic de syndrome de VKH dans sa forme purement oculaire, forme dite ‘'possible’‘ a été retenu. Une corticothérapie en bolus de méthylprednisolone de 1g/j pendant 3 jours avec relais par voie orale de 1mg/kg/j de prednisone substitué au troisième mois par un traitement par l'azathioprine. L’évolution à été marquée par la récupération fonctionnelle presque complète en 2 mois avec disparition des DSR à l'angiographie et persistance de quelques zones hyperfluorescentes correspondant trouble de pigmentation de l’épithélium pigmentaire.

## Discussion

Les critères [1] de 2001 ont permis d'affiner le diagnostic du syndrome de Vogt-Koyanagi-Harada. Cependant ces critères diagnostiques sont semblables à une photographie instantanée de la maladie et ne rendent pas compte de la cinétique d'apparition des symptômes qui peuvent être espacés dans le temps. Il est important d'adapter les outils diagnostiques au stade clinique de la maladie.

Ainsi, au stade aigu, les signes neurologiques [2] et ORL sont les plus fréquemment rapportés et la ponction lombaire sera le plus souvent anormale, même en l'absence de toute plainte fonctionnelle. Au stade chronique, les signes dermatologiques et la dépigmentation du fond d’œil sont les signes les plus fréquemment observés. En première intention, les données de l'examen clinique, complétées par la ponction lombaire et l'angiographie à la fluorescéine permettent de poser le diagnostic de syndrome de Vogt-Koyanagi-Harada dans plus de 98% des cas. En cas de doute, l’échographie [3], l'angiographie ICG, et l'OCT peuvent s'avérer utiles en seconde intention pour préciser l'atteinte oculaire, tandis que l'IRM cérébrale et l'audiogramme compléteront le bilan neurologique. Ces examens aident à écarter les autres pathologies choroïdiennes (L'ophtalmie sympathique, épithéliopathie en plaque, le lymphome intra oculaire, syndrome des taches blanches évanescentes…).

Le syndrome de VKH dans sa forme dite possible reste rare et ne représente que 10% des formes cliniques [4]. C'est une affection sévère mettant en jeu le pronostic visuel malgré les traitements agressifs. Sa sévérité réside dans la fréquence des récidives [5], dans la corticorésistance et dans la forte incidence des complications à type de cataracte, glaucome, atrophie épithéliale qui conditionnent le pronostic et expliquent les acuités visuelles finales médiocres. La prise en charge doit être précoce afin d′essayer d′améliorer le pronostic visuel.

## Conclusion

Le syndrome de VKH dans sa forme dite possible est rare mais dont le diagnostic doit etre évoqué devant les aspects caractéristiques au fond d’œil et à langiographie en vue d'un traitement précoce conditionnant le bon pronostic.
